# Progress in understanding the role and mechanism of miRNAs in osteoporosis

**DOI:** 10.3389/fendo.2025.1544944

**Published:** 2025-08-19

**Authors:** Feifei Meng, Changwei Yang, Na Li, Huaxin Wang

**Affiliations:** ^1^ Shandong University of Traditional Chinese Medicine, Jinan, Shandong, China; ^2^ College of First Clinical Medicine, Shandong University of Traditional Chinese Medicine, Jinan, Shandong, China

**Keywords:** osteoporosis, bone remodeling, miRNAs, mechanism, exosomes

## Abstract

Osteoporosis is a systemic metabolic disorder characterized by compromised bone strength and increased fracture risk. Exosomes, extracellular vesicles measuring 40–160 nm in diameter, are critical mediators of intercellular communication. Among their bioactive components, microRNAs (miRNAs) have garnered attention for their role in the pathogenesis of Osteoporosis. Through complementary binding to the 3′ untranslated regions of target genes, miRNAs regulate key processes such as bone formation, bone resorption, angiogenesis, and bone immunity. This review provides a comprehensive summary of the regulatory roles and underlying mechanisms of miRNAs in osteoporosis, offering insights into potential therapeutic strategies.

## Graphical Abstract

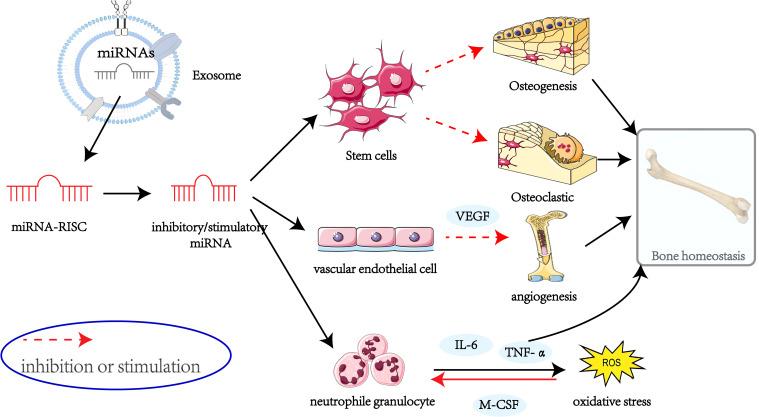

## 1 Introduction

Osteoporosis (OP) is a metabolic bone disease characterized by reduced bone mass and deterioration of bone microarchitecture, resulting in increased bone fragility and susceptibility to fractures (1, 2). In recent years, OP has become increasingly prevalent among the elderly, contributing to greater fracture risk, diminished quality of life, and increased mortality in severe cases (3). According to the International Osteoporosis Foundation, osteoporosis imposes a substantial global burden, with approximately 1 in 3 women and 1 in 5 men aged over 50 experiencing osteoporotic fractures (4). Using the WHO definition, OP affects approximately 6.3% of men and 21.2% of women over 50 globally, suggesting approximately 500 million people may be affected (5). These trends underscore the urgent need to address OP as a significant global public health challenge, especially in the context of an ageing population.

Exosomes are double-layered lipid membrane vesicles, 40–160 nm in diameter, produced within the endosomal compartments of most eukaryotic cells, and primarily contain biomolecules such as proteins, RNAs, and lipids that play significant roles in intercellular communication (6, 7). MicroRNAs (miRNAs), critical epigenetic regulators, participate in bone development, homeostasis, and repair processes by modulating the differentiation and activity of osteoblasts and osteoclasts, and are strongly linked to osteoporosis pathogenesis (8, 9). miRNA expression is regulated at multiple levels, including epigenetic mechanisms such as DNA methylation and histone modifications, as well as proteins regulating their maturation. Furthermore, miRNA expression is significantly influenced by environmental factors such as diet (e.g., vitamin D), exercise, pharmaceuticals, hormones, smoking, and even circadian rhythms, which alter their circulating levels (8). It has been found that miRNAs, as important active components of exosomes, can be transported to recipient cells via exosomes, thereby affecting the post-transcriptional expression of target genes and regulating the life activities of recipient cells (10, 11). Increasing evidence indicates that exosomal miRNAs may exert influence over the skeletal microenvironment by regulating gene expression through post-transcriptional gene silencing, which is either directly or indirectly implicated in the bone remodeling process (12–14). Several studies have reviewed the importance of miRNAs in the pathobiology of human disease (15, 16). miRNAs, acting upstream in the gene expression pathway, exhibit changes in circulating levels that can earlier reflect biological effects in the skeletal system, thus making them more sensitive potential biomarkers than classical protein biomarkers (8). The aim of this review is to provide an overview of the role of exosomal miRNAs in bone remodeling and their regulatory mechanisms in OP.

## 2 The biogenesis of miRNAs

**Figure 1:**
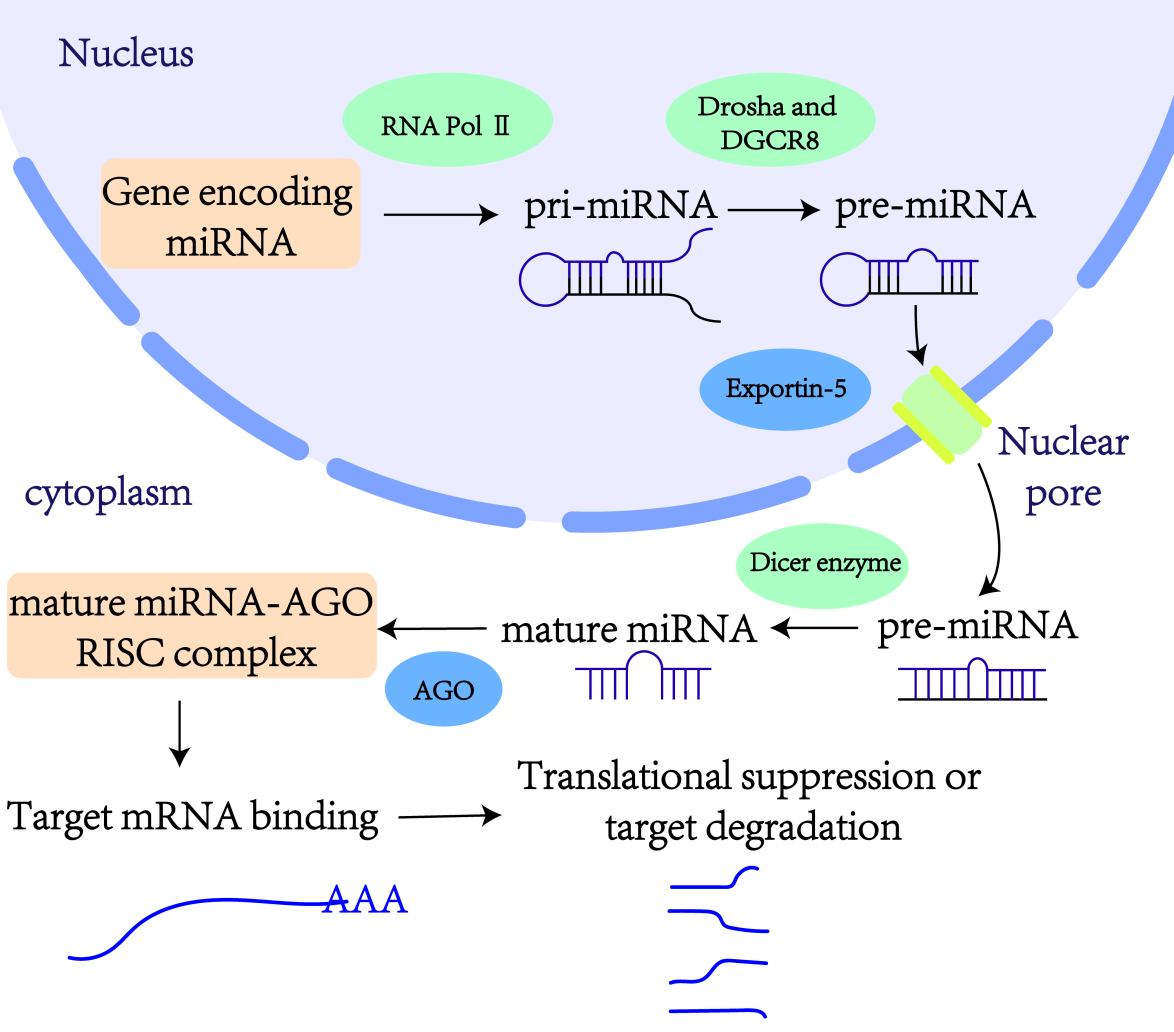
Production and action pathway of exosomal miRNAs.

miRNAs are small endogenous non-coding RNA molecules, typically 19–25 nucleotides in length (17). In 1993, Victor Ambros and colleagues first identified the lin-4 gene as being involved in the developmental regulation of the nematode *Caenorhabditis elegans* (18). This discovery marked lin-4 as the first member of the miRNA family. Generally, miRNA genes are transcribed into primary miRNAs (pri-miRNAs) by RNA polymerase II. These pri-miRNAs are subsequently processed into precursor miRNAs (pre-miRNAs) by the nucleases Drosha and its cofactor DGCR8 (19). Pre-miRNAs are then transported from the nucleus to the cytoplasm by the Exportin-5 complex. In the cytoplasm, the enzyme Dicer cleaves pre-miRNAs, yielding mature double-stranded miRNAs. These mature miRNAs are loaded onto the Argonaute protein, forming the miRNA-induced silencing complexes (19). Within these complexes, one strand of the miRNA duplex is rapidly degraded, while the other strand—the functional mature miRNA—bind to the 3’ untranslated regions (3’ UTRs) of target mRNAs (**Figure 1**). This binding regulates gene expression post-transcriptionally by either inhibiting translation or inducing degradation of the target mRNAs. Thus, miRNAs play critical roles in various cellular biological processes.

The functional role of miRNAs primarily depends on their ability to bind to the 3’ UTRs of target mRNAs, thereby regulating gene expression (20). Numerous studies have demonstrated that miRNAs are widely distributed across various human tissues and organs, significantly affecting essential biological processes such as proliferation, apoptosis, and differentiation (21, 22). In addition to their roles in normal physiology, miRNAs are pivotal in various pathological conditions, such as cancer, pulmonary fibrosis, and diabetes (23). Notably, the expression levels of specific miRNAs can be modulated to regulate target gene expression, thus influencing normal cellular activities (24). Furthermore, miRNAs function within regulatory networks, as individual miRNAs may target multiple genes, and individual genes can be regulated by multiple miRNAs, highlighting the complexity of their regulatory roles (25).

## 3 The exosomal miRNAs linked to OP

OP, a prevalent and serious bone disease, is characterized by reduced bone mineral density and increased susceptibility to fractures. It primarily results from an imbalance between osteoblast-mediated bone formation and osteoclast-mediated bone resorption (26). However, the pathogenesis of OP extends beyond bone remodeling disruptions to involve factors such as estrogen deficiency, oxidative stress, and inflammation (27, 28).

Emerging evidence indicates that dysregulated microRNA (miRNA) expression can significantly influence osteoblast differentiation and activity, contributing to bone remodeling imbalances and the progression of OP (29). The multifaceted nature of OP underscores the need to comprehensively understand its underlying mechanisms and to identify novel therapeutic targets.

**Figure 2:**
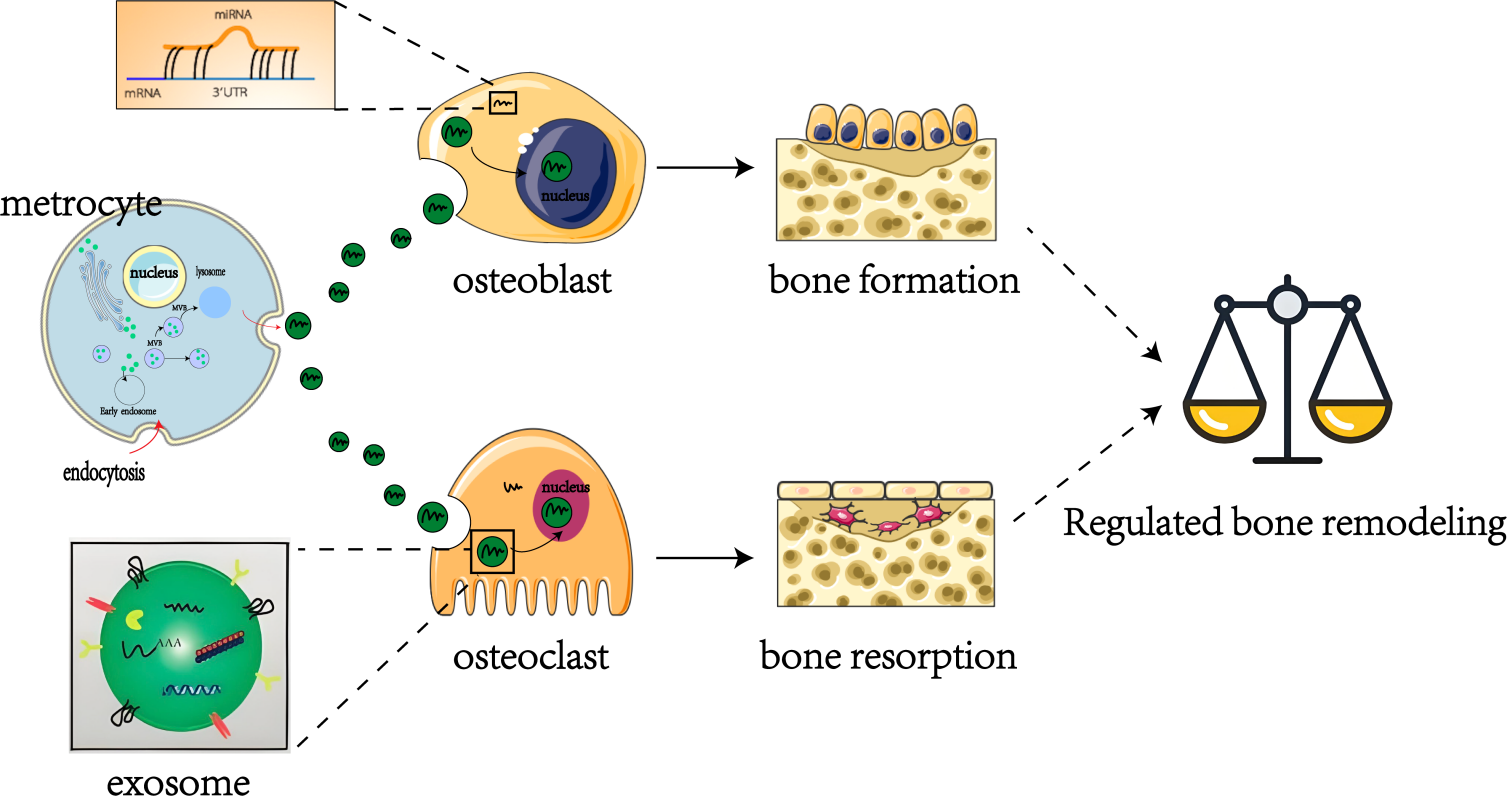
Regulation of exosome miRNAs in bone remodeling.

In recent years, the regulatory roles of miRNAs in OP - particularly those encapsulated within exosomes - have attracted considerable attention (30–32). These miRNAs play critical roles in modulating osteoblast and osteoclast proliferation and differentiation, thereby maintaining the delicate equilibrium between bone formation and resorption (**Figure 2**) (33). Bioinformatics analyses have revealed distinct miRNA expression patterns associated with postmenopausal osteoporosis (34). Notably, a comparative study of patients with osteoporotic versus non-osteoporotic hip fractures identified five miRNAs significantly elevated in the serum and bone tissue of osteoporotic patients (35). Additionally, numerous studies have highlighted the pivotal role of exosomal miRNAs in OP pathogenesis (36–38). For example, research investigating miRNA levels in serum samples from postmenopausal women with osteoporosis identified 331 differentially expressed miRNAs, including 122 upregulated and 209 downregulated miRNAs compared to controls (39). Collectively, these findings illustrate the intricate relationship between miRNAs and OP pathogenesis, highlighting potential therapeutic targets. This review therefore aims to further elucidate these miRNA-disease connections and facilitate the development of innovative treatment strategies.

## 4 The regulatory roles of miRNAs in OP

### 4.1 Role of miRNAs in regulating bone formation

Bone formation is a critical process within bone remodeling, involving the transformation of bone marrow mesenchymal stem cells (BMSCs) into osteoblasts. These osteoblasts are essential for secreting collagen fibers and facilitating bone matrix mineralization (40). Exosomal miRNAs, as remarkable regulators, fine-tune the expression of factors associated with bone formation, guiding osteoblast differentiation and function while influencing the intricate process of bone reconstruction (41). Dysregulated miRNA expression has been identified as a significant pathological factor impairing bone formation. miRNAs regulate osteogenic differentiation and bone formation through key signaling pathways, including the transforming growth factor β (TGF-β)/bone morphogenetic protein (BMP) pathway, the Wingless/Int-1 (Wnt)/β-catenin pathway, and the Notch signaling pathway (42).

Consistent with these roles, accumulating evidence highlights the sophisticated role of miRNAs in orchestrating osteogenesis (**Table 1**). miRNAs achieve this by either regulating key transcription factors through complex signaling cascades or directly targeting osteoblast proliferation and differentiation (43), emphasizing their potential as crucial mediators in bone health and promising therapeutic targets for bone-related disorders.

**Table 1:** miRNAs that play a key role in bone formation.

miRNAs	Target genes or pathways	Bone formation	References
miR-23a	Runx2	(-)	(46)
miR-30c	Runx2	(-)	(46)
miR-34c	Runx2	(-)	(46)
miR-133a	Runx2	(-)	(46)
miR-135a	Runx2	(-)	(46)
miR-137	Runx2	(-)	(46)
miR-204	BMP-2/Runx2	(-)	(46, 50)
miR-205	Runx2	(-)	(46)
miR-217	Runx2	(-)	(46)
miR-218	Runx2	(+)	(46)
miR-338	Runx2	(-)	(46)
miR-30a-5p	Runx2	(-)	(51)
miR-338-3p	Runx2	(-)	(52)
miR-150-3p	Runx2	(+)	(53)
miR-1275	Runx2	(+)	(54)
miR-21	Smad7	(+)	(55)
miR-98	BMP-2	(-)	(57)
miR-153	BMP-2	(-)	(58)
miR-214	BMP-2	(-)	(59)

(-) on behalf of the negative control; (+) represents positive regulation.

The Runt-related transcription factor (Runx) family is highly conserved and plays a vital role in organ development, cell metabolism, and stem cell differentiation (44). This family includes Runx1, Runx2, and Runx3, with Runx2 being essential for bone development by regulating osteoblast-mediated bone formation via various signaling pathways (45). Zhang et al. identified a set of 11 Runx2-targeted miRNAs, including miR-23a, miR-30c, miR-34c, miR-133a, miR-135a, miR-137, miR-204, miR-205, miR-217, miR-218, and miR-338, which exhibit lineage-specific expression patterns in mesenchymal cells. Among these, all except miR-218 showed a negative correlation with Runx2 expression (46). Most of these miRNAs have been previously shown to influence osteogenic differentiation (47–50). Notably, Zhang et al. also identified Runx2 as a downstream target of miR-30a-5p, where long non-coding RNA (lncRNA)-XIST promoted osteogenic differentiation in BMSCs by competitively binding miR-30a-5p and subsequently upregulating Runx2 expression (51). Additionally, Liu et al. observed increased miR-338-3p expression in an ovariectomy-induced rat osteoporosis model, reporting that miR-338-3p inhibited osteogenic differentiation by targeting both Runx2 and fibroblast growth factor receptor 2 (FGFR2) (52). Moreover, studies have demonstrated that specific exosomal miRNAs (e.g., miR-150-3p, miR-1275, and miR-21) enhance osteoblast differentiation and bone formation by upregulating Runx2 expression (53–55). These findings underscore the critical regulatory network involving Runx2 and miRNAs in bone biology.

Bone morphogenetic protein 2 (BMP-2) plays a crucial role in guiding mesenchymal stem cells (MSCs) towards becoming osteoblasts. BMP-2 achieves this by interacting with enzyme receptors on target cells, regulating the Smad signaling pathway, and ultimately activating osteogenic genes, thereby facilitating the formation of new bone (56). Studies have demonstrated that miRNAs such as miR-98 and miR-153 inhibit osteoblast proliferation and differentiation by directly targeting BMP-2, consequently influencing bone formation processes (57, 58). In human bone marrow mesenchymal stem cells (hBM-MSCs), miR-214 down-regulates the binding of BMP-2 expression to 3’ UTRs, and silencing miR-214 enhances osteogenic differentiation (59). Similarly, miR-204’s direct interaction with BMP-2 mRNA significantly impairs the differentiation of rat bone marrow MSCs (50). Maintaining a balance between osteogenic and adipogenic differentiation of MSCs is crucial for bone homeostasis (60). Inhibiting adipogenic differentiation, thus favoring osteogenic differentiation, represents a strategy to mitigate bone loss and enhance bone mass. MiR-146b-5p inhibits adipogenic differentiation of BMSCs from children with aplastic anemia by targeting SIAH_2_ and reducing PPARγ stability (61). Furthermore, scholars have found that miR-140 expression is downregulated in the serum of neonatal patients with developmental dysplasia, implying a potential role in neonatal bone formation, although its specific mechanism remains unreported (62).

### 4.2 Role of miRNAs in regulating bone-resorption

Previous research has demonstrated that the receptor activator of nuclear factor κB ligand (RANKL)/receptor activator of nuclear factor κB (RANK)/osteoprotegerin (OPG) signaling pathway plays a pivotal role in regulating bone metabolism. RANKL, a key initiator of osteoclast differentiation, promotes the transformation of macrophages into osteoclasts by stimulating the expression of the transcription factor nuclear factor of activated T-cells cytoplasmic 1 (NFATc1). In contrast, OPG acts as a decoy receptor, binding to RANKL and thus inhibiting the RANKL/RANK interaction, which mitigates osteoclast activation and differentiation (63–65).

A study has demonstrated that the overexpression of miR-503 in ovariectomized mice directly inhibits the RANKL/RANK signaling pathway, reducing osteoclast activity (66). Several microRNAs, such as miR-124, regulate osteoclast differentiation by targeting NFATc1 mRNA. Specifically, miR-124 suppresses NFATc1 expression, affecting osteoclast differentiation through both RANKL-dependent and RANKL-independent pathways (67). In another study, Li et al. identified NFATc1 as a direct target of miR-193-3p. Overexpression of miR-193-3p inhibited NFATc1 expression, leading to reduced bone resorption in ovariectomized (OVX) mice (68). Additionally, recent research revealed that lncRNA-MIRG, acting as a competing endogenous RNA for miR-1897, inhibits miR-1897 expression. This inhibition enhances NFATc1 expression, promoting osteoclast differentiation from monocytic macrophages and exacerbating bone resorption in osteoporosis patients (69). Experimental data confirmed that increased miR-21 expression correlates with higher RANKL levels, reduced OPG concentrations, an increased RANKL/OPG ratio, and accelerated bone resorption, ultimately contributing to the progression of osteoporosis (70). Furthermore, another study demonstrated that knocking down miR-21 in mice reduced osteoclast function and number, resulting in increased trabecular bone volume and decreased bone resorption (71). These findings underscore the critical regulatory role of miRNAs in the RANKL/RANK/OPG signaling pathway and their potential as therapeutic targets in OP management. Moreover, miR-27a modulates estrogen-related processes by targeting the expression of PPARγ, thereby inhibiting osteoclast differentiation and bone resorption (72). Similarly, miR-146a has been identified as a key regulator in osteoclast formation. Deletion of miR-146a impairs osteoclast-mediated bone resorption, offering protection against OVX-induced bone loss (73). Together, these findings highlight the therapeutic potential of exosomal miRNAs for targeting osteoclast function in osteoporosis treatment. Future research is essential to further explore miRNA-dependent pathways that regulate osteoclast function, offering deeper insights into their role in bone diseases and paving the way for novel therapeutic strategies (**Table 2**).

**Table 2:** miRNAs that play a key role in bone resorption.

miRNAs	Target genes or pathways	Bone resorption	References
miR-124	NFATc1	(-)	(67)
miR-193-3p	NFATc1	(-)	(68)
miR-1897	NFATc1	(-)	(69)
miR-21	OPG	(+)	(70)
miR-27a	PPARγ	(-)	(72)
miR-146	/	(+)	(73)
miR-503	RANKL/RANK pathway	(-)	(66)

### 4.3 Role of miRNAs in regulating angiogenesis

Bone formation and angiogenesis are closely interconnected processes. Angiogenesis plays a critical role in promoting bone formation and maintaining bone homeostasis, particularly during bone development and fracture healing, where angiogenesis and osteogenesis are coupled (74). Vascular endothelial growth factor (VEGF) secreted by osteoblasts is an important regulator of the coupling of osteogenesis and angiogenesis; in particular, VEGFA, as a major pro-angiogenic factor, can attract endothelial cells to bone tissue and directly regulate the differentiation of osteoblasts and osteoclasts, thereby affecting bone metabolism (75). Dysregulated miRNA expression can lead to abnormal angiogenesis, therefore miRNAs can be used as potential targets to regulate angiogenesis and thus participate in the regulation of bone remodeling.

Several studies have shown that VEGFA plays an important regulatory role in OP (76, 77). Duan et al. found that osteogenic differentiation was decreased by miR-16 upregulation and increased by miR-16 downregulation (78). To further determine the regulatory role of miR-16 in OP, Yu et al. identified miR-16-5p as a potential miRNA targeting VEGFA mRNA using TargetScanHuman and DIANA software, and found that miR-16-5p can able to inhibit osteogenic differentiation by downregulating VEGFA expression (79). In addition, both miR-214-3p and miR-195 were found to be negative regulators of angiogenesis (80, 81). Differently, miR-214-3p was able to inhibit angiogenesis by downregulating VEGF expression and releasing negatively regulated angiogenic signals, whereas miR-195 inhibited bone-derived differentiation and angiogenesis in MSCs by decreasing the paracrine effect of MSCs on angiogenesis. Similarly, miR-181c-5p, an anti-angiogenic miRNA, is involved in regulating bone remodeling process by targeting and regulating the expression of Frizzled-related protein-1 (SFRP1), a negative regulator of osteoblasts, and activating the Wnt/β-catenin signaling pathway (82–84).

In addition, there is evidence that miR-29a, miR-126 and miR-136-3p all have positive effects on angiogenesis (85–87). Among these, miR-29a can effectively promote angiogenesis and osteogenesis in mice, while miR-126 and miR-136-3p are able to promote angiogenesis to accelerate the process of bone formation by triggering the production of a response signal in human umbilical vein endothelial cells (HUVEC), providing a new therapeutic for OP. These miRNAs represent promising therapeutic targets for the treatment of osteoporosis (**Table 3**).

**Table 3:** miRNAs that play a key role in angiogenesis.

miRNAs	Target genes or pathways	Angiogenesis	References
miR-16	VEGFA	(-)	(79)
miR-214-3p	VEGF	(-)	(80)
miR-195	VEGF	(-)	(81)
miR-181c-5p	SFRP1/Wnt pathway	(+)	(84)
miR-29a	/	(+)	(85)
miR-126	SPRED1/Ras/Erk signaling pathway	(+)	(86)
miR-136-3p	PTEN	(+)	(87)

### 4.4 Role of miRNAs in regulating osteoimmunology

OP is increasingly recognized as an inflammatory bone disease characterized by a close interplay between immune cells and skeletal tissues (88, 89). In addition to inflammatory cytokines such as interleukin-6 (IL-6), tumor necrosis factor-α (TNF-α) and macrophage colony-stimulating factor (M-CSF), immune cells produce high levels of reactive oxygen species (ROS), which activate osteoclastogenic bone resorption (90). ROS are a major cause of oxidative stress (OS), which exacerbates injury (91). Taken together, inflammatory cytokines and immune cell-derived ROS interact directly or indirectly with osteoblasts, leading to an inflammatory response that drives the development of OP and regulates communication between the skeletal and immune systems (**Table 4**).

**Table 4:** miRNAs that play a key role in osteoimmunology.

miRNAs	Target genes or pathways	Osteoimmunology	References
miR-223-3p	IL-6	(+)	(92)
miR-122	TNF-α	(+)	(98)
miR-146a	M-CSF	(-)	(73)
miR-21	M-CSF	(+)	(99)
miR-143-3p	M-CSF	(+)	(100)
miR-125a-5p	ROS, VEGF	(-)	(103)
miR-424	FGF2	(+)	(105)

Cheng et al. found that IL-6 is a direct target gene of miR-223-3p, which inhibits the persistent pro-inflammatory response by suppressing IL-6 expression, thereby improving the bone microenvironment and regulating bone metabolism (92). Other studies have shown that miRNAs such as miR-495, miR-200c, miR-146a, miR-27a can promote bone formation by directly or indirectly down-regulating IL-6 (93–96). TNF-α inhibits osteoclast activity and stimulates osteoblast proliferation and differentiation at certain stages of differentiation (97), an effect which is improved by transfection of miR-122 mimics, reducing TNF-α stimulation in the organism, and reducing apoptosis (98). In addition, M-CSF acts as a regulator of osteoclasts and is able to induce osteoclast differentiation. miR-21, miR-143-3p and inhibition of miR-146a reduce osteoclast activity and inhibit osteoclast differentiation by reducing the amount of M-CSF in the bone microenvironment, thereby reducing bone loss (73, 99, 100).

Moreover, oxidative stress and exosome-derived miRNAs significantly influence OP pathogenesis (101, 102). Ye et al. found that miR-125a-5p and ROS were upregulated during osteogenic induction of hADSCs *in vitro*, suggesting that miR-125a-5p may reduce osteoblasts by exacerbating ROS damage and inhibiting VEGF expression, thereby reducing osteoblast sexual bone formation (103). Notably, forkhead box O1 (FoxO1), an important protein that protects bone from oxidative damage, is able to inhibit osteogenic differentiation by reducing ROS levels in cells (104). Furthermore, FoxO1 inhibited miR-424 expression and promoted cell proliferation and osteogenic differentiation, in part through the miR-424/FGF2 pathway (105).

## 5 Discussion

OP is a complex systemic metabolic disease characterized by multiple interacting mechanisms and pathways, as well as intricate communication between mesenchymal stromal cells, immune cells, and other biological cell types. This communication occurs either through direct cell-to-cell contact or via secreted factors, which are often transported by extracellular vesicles such as exosomes. Current treatments for OP primarily focus on inhibiting osteoclast proliferation and activation to reduce the rate of bone resorption. However, although drugs like bisphosphonates, denosumab, and estrogens are commonly used, long-term administration of these agents is associated with significant adverse effects and limited efficacy; moreover, the fundamental pathophysiological mechanisms of osteoporosis are not yet fully understood (106). As a result, there is a pressing need for further research into the molecular mechanisms regulating bone metabolism. Identifying low-toxicity, highly efficient drug targets that promote bone health could provide innovative strategies and methods for the prevention and treatment of OP.

miRNAs have the potential to serve as an early diagnostic biomarker for OP as well as a means of detecting the progression of this disease (64). Utilizing miRNAs that regulate osteoporosis pathogenesis could represent an effective therapeutic approach. For instance, miR-375 has been identified in the serum of postmenopausal women with an elevated risk of osteoporosis, serving as a potential marker of disease progression (107). Furthermore, prolonged administration of bisphosphonates has been associated with inhibited bone formation due to the overexpression of miR-30a-5p (108). It is noteworthy that, due to their multi-pathway and multi-target regulatory ability, the same miRNAs may regulate different targets, and multiple miRNAs may regulate the same or different mechanisms through the same or different targets. Consequently, the identification of specific miRNAs and their molecular targets and regulatory mechanisms involved in bone metabolism is an essential preliminary step in the development of clinical applications.

**Figure 3:**
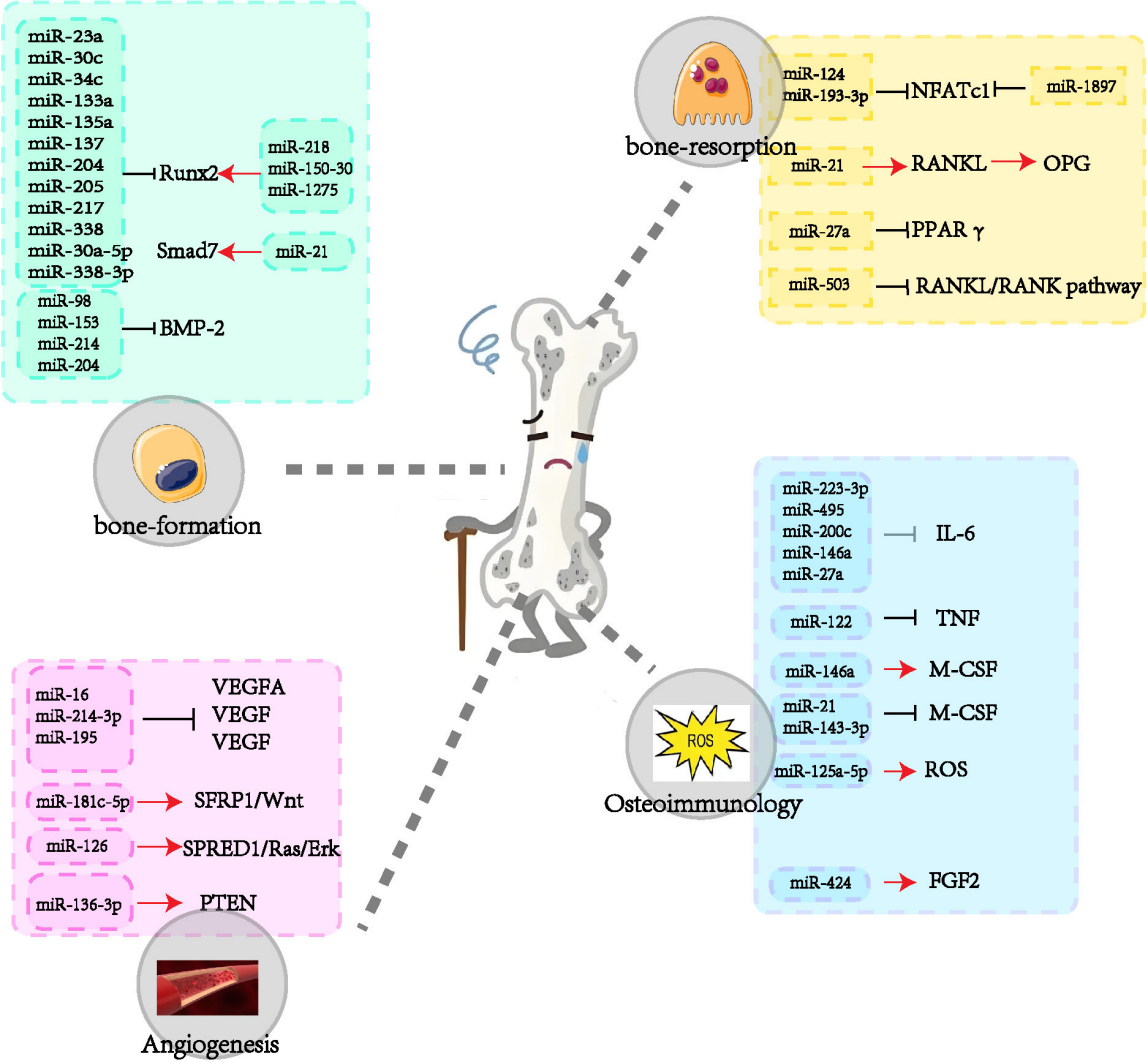
The role of miRNAs in osteoporosis.

The role of miRNAs in exosomes in the aetiology and progression of OP has become a focus of research in recent years. These miRNAs regulate the proliferation and differentiation of osteoblasts and osteoclasts, influence angiogenesis, and participate in processes such as bone immunology. The present review provides a comprehensive overview of the role of multiple miRNAs in the regulation of osteoporosis genesis mechanisms (**Figure 3**). However, as there is no one-to-one correspondence between miRNAs and genes, specific miRNAs may affect multiple genes, which may result in potential side effects. Consequently, miRNA-based pharmaceutical agents for the management of osteoporosis have yet to be subjected to clinical trials.

The discovery of miRNAs and their regulatory roles in bone metabolism is closely linked to the development of new diagnostic and therapeutic techniques for OP. To translate these insights into clinical practice, comprehensive basic and clinical research is required to develop novel and more effective osteoporosis treatments. In the era of precision medicine, exploring exosomal miRNAs and their functions offers a unique approach to unraveling the molecular mechanisms of OP. miRNAs, as the main active components secreted by exosomes, have the unique advantage of being fine and precise, which will make this a reliable, sensitive and advanced technology for the treatment of OP in future studies.
